# Cast accuracy obtained from different impression techniques at different implant angulations (in vitro study)

**DOI:** 10.1186/s40729-018-0118-6

**Published:** 2018-03-20

**Authors:** Enas A. Elshenawy, Ahmed M. Alam-Eldein, Fadel A. Abd Elfatah

**Affiliations:** 1 Dental Biomaterials Department, Faculty of dentistry, Tanta University, Tanta, Egypt; 2 Prosthodontic Department, Faculty of dentistry, Tanta University, Tanta, Egypt

**Keywords:** Direct technique, Indirect technique, Internal connection implant, Splinting procedure

## Abstract

**Background:**

Angulated implants may result in inaccurate impressions, and the impression technique may affect the accuracy of the definitive cast. This study was designed to compare the dimensional accuracy of casts obtained from three impression techniques for three definitive lower casts with implants at different angulations.

**Methods:**

Three Osseolink implants were placed in three reference models with different angles (parallel, 15° and 30°). Impressions of each model were made with three techniques (*n* = 10 per group): indirect, unsplinted direct, and acrylic resin-splinted direct technique. Impressions were poured with type IV dental stone. Inter-implant distances were measured for casts using a coordinate measuring machine, and the deviations from the reference models (Δ*r*) were calculated. Data were analyzed using one-way ANOVA followed by post hoc tests to detect significance between groups (*α* = 0.05).

**Results:**

This study showed that the deviations in micrometers from the reference model were the least for acrylic resin-splinted direct technique (Δ*r*1 = 49.96, Δ*r*2 = 50.36) versus indirect (Δ*r*1 = 93.8, Δ*r*2 = 90.9) and unsplinted direct techniques ((Δ*r*1 = 67.07, Δ*r*2 = 68.66) in 30° angulated implant situation (*p* value < 0.0001^*^ for both Δ*r*1 and Δ*r*2). In 15° angulated implants, both the acrylic resin-splinted direct (Δ*r*1 = 44.64, Δ*r*2 = 45.58) and unsplinted direct techniques (Δ*r*1 = 47.39, Δ*r*2 = 55.28) were more accurate than indirect technique (Δ*r*1 = 64.8, Δ*r*2 = 68.3) (*p* value < 0.0001^*^ for both Δ*r*1 and Δ*r*2). While in parallel condition, no difference was found between all three techniques (*p* value = 0.085, 0.056 for Δ*r*1 and Δ*r*2, respectively).

**Conclusions:**

The impression technique affected the accuracy of definitive casts. The acrylic resin splinted direct technique produced the most accurate casts, followed by direct unsplinted and indirect techniques. Furthermore, implant angulation affected the impression accuracy. When implant angulation increased from parallel implants to 30°, the forces of deformation increased, which resulted in increased distortion.

## Background

Precise working casts are essential to fabricate passively fitting implant prostheses. Accurate implant impressions play a significant role and serve as a starting point in the process of producing good working casts [1]. Thus, the comparative accuracy of the impression techniques becomes a significant issue in consideration of passive fit. An inaccurate impression may result in prosthesis misfit, which can lead to further problems such as mechanical and/or biological complications [2].

Impression technique, type of impression material [3], splinting or non-splinting impression copings, type of splinting material, and number and angulation of implants [4] are the factors that affect the accuracy of impression.

Two main implant impression techniques are used for transferring the intra-oral spatial relationship of the implants to the working cast. One impression technique is the direct open tray technique that uses a custom tray with windows exposing the impression copings. The other impression technique is the indirect technique that uses closed tray [5]. With the direct technique, both splinting and non-splinting of impression copings to improve the accuracy of impressions have been advocated [6].

In the open tray technique, the impression coping is incorporated in the impression and is removed from the mouth together with the set impression [1]. In the closed tray technique, the impression copings are retained in the mouth when the set impression is removed, and then, these copings are unscrewed from the mouth and connected to the implant analogs. This coping-implant analog assembly is repositioned into its respective position within the impression [7].

To ensure maximum accuracy, some authors emphasized the importance of splinting impression copings together intraorally before making an impression and some authors sectioned the splint material leaving a thin space and then rejoining with a minimal amount of the same material to minimize polymerization shrinkage. However, inconsistent results have been obtained [8, 9].

The implant impression can be at the abutment or implant level. The implant level impression is preferred in the esthetic zones and reduces the number of treatment visits. However, it presents unique challenges to the prosthodontist and errors can be introduced in many ways due to a rotation error that occurs when implants are connected to impression copings and to dislodgement of the impression material during removal of the impression tray from the mouth [10].

The adoption of tilted implants for the rehabilitation of both edentulous mandibles and maxillae has been proposed in the recent years. In the mandible, tilting of the distal implants may prevent damage to the mandibular nerve. Implants of conventional length can be placed, allowing engagement of as much cortical bone as possible, thus increasing primary stability [11]. However, the lack of parallelism between implants may result in increased distortion of impression material during removal from the mouth that may generate an inaccurate model [12–14].

Several impression materials have been used for multiunit implant impression; the most commonly described were addition silicone and polyether impression materials. This can be correlated to their improved accuracy [7]. Polyvinylsiloxanes show the smallest dimensional changes in comparison to the other elastomeric impression materials since they do not produce a volatile by-product during polymerization [15, 16].

This study was conducted to evaluate the effect of impression techniques and implant angulations on the accuracy of impressions in parallel and angulated implants in three mandibular models simulating clinical situations.

Three null hypotheses were tested:

1. There is no significant difference in impression accuracy whether an indirect, direct unsplinted, or direct acrylic resin splinted impression techniques were used.
2. There is no significant difference in impression accuracy whether implants had a 0°, 15°, or 30° angulation to a reference line perpendicular to the cast.
3. There is no significant interaction between the impression technique and implant angulation.

## Method

### Master model fabrication

Three epoxy resin (Ramses medical products factory, Alex, Egypt) completely edentulous mandibular models representing a clinical situation were used as definitive casts. Each cast had three implants (OsseoLink USA LLC. 4 mm × 9 mm, internal connection type) arranged with one implant at the midline and the other two implants at the premolar regions.

- *Cast (1)* had all the three implants parallel to each other and perpendicular to the plane of the cast.
- *Cast (2)* had implant at the midline perpendicular to the plane of the cast and implants at the premolar regions angulated at 15° to a line drawn perpendicular to the occlusal plane.
- *Cast (3)* had implant at the midline perpendicular to the plane of the cast and implants at the premolar regions angulated at 30° to a line drawn perpendicular to the occlusal plane.

Each definitive cast was held in a vertical milling machine (Milling & Drilling machine, RF-Sakkary, Taiwan), and a protractor was used to align the cutting bur in the proper angulation by tilting the machine table.

The implants were placed in each definitive cast with a hand wrench and were numbered as follows: the middle implant was number 1, the left premolar implant was number 2, and the right premolar implant was number 3; and this numbering was used throughout the study.

### Custom tray fabrication

#### Preparation of stone duplicate for each model

After the impression copings were connected to the definitive models, the space for impression material was created with baseplate wax (Cavex Setup Waxes, Haarlem, Holland). Stoppers were made on the molar regions to standardize the tray position.

An impression was taken from each model, using condensation silicone (Zetaplus, Zhermack SpA, Italy). Impressions were boxed and poured with type IV dental stone (Elite® Stone, Zhermack GmbH Deutschland) in a vacuum device. The resulting three stone casts were used to fabricate the custom trays.

#### Preparation of the master custom tray

Self-cured acrylic resin (Acrostone cold cure special tray material, Cairo, Egypt) was used to make the master custom trays. There are two master trays for each cast: one closed tray for the indirect technique and one with three windows for the direct unsplinted and acrylic resin-splinted technique.

#### Preparation of replicate custom trays

Dental flask was used for fabricating replicate trays from each master tray using the master custom tray and type IV dental stone to fabricate a two-part mold to make 30 custom trays for each cast, ten closed and twenty open trays.

The trays were made of self-cured acrylic resin (Acrostone cold cure special tray material, Cairo, Egypt). The trays were perforated for added retention of the impression material. Tray handles were made and attached to the custom trays. The trays were stored at the room temperature for 24 h before impression taking.

### Impression procedure

Three different groups of implant level impression techniques were made (*n* = 10 per group) for each reference cast, a total of nine subgroups.

Addition silicone impression material (Enthus PVS Impression Material, Dharma Research, USA) with medium consistency was used for all impression procedures.

The impression procedure was standardized as follows:

1. A 1.5 kg metal block exerted a standardized pressure on each tray during the polymerization.
2. The copings were secured to the implants using dedicated torque wrench calibrated at 10 Ncm.
3. Tray adhesive was painted on the trays before making impressions.
4. The impression material was mixed using an impression gun.
5. The reference models were painted with separating medium before the impression procedures to simulate oral condition.

#### In the indirect technique

**Fig. 1:**
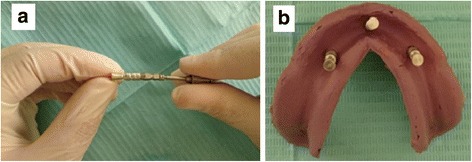
**a** Attaching the analog to the coping using the screw driver. **b** Impression after insertion of the coping-analog till hearing the audible click

Closed tray impression copings remained on the definitive cast after removal of the impression. These impression copings were removed one at a time from the definitive cast and attached to an implant analog. The combined impression coping analog unit was inserted into the impression by firmly pushing it into place to full depth and slightly rotating clockwise to feel for the anti-rotational resistance (Fig. 1 a, b).

#### In the direct unsplinted technique

**Fig. 2:**
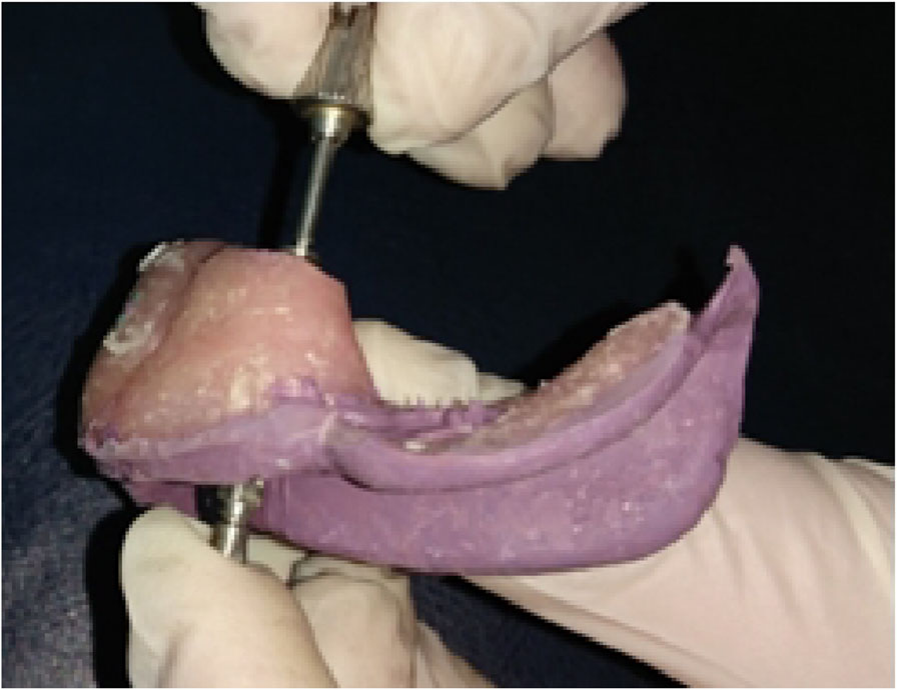
Connecting the analog with the coping using the guide pin

The guide pins were loosened with a hex driver and removed, and the tray was separated with the impression copings locked in the impression. The guide pins were placed back into the impression copings from the top, while an implant analog was connected to the hex on the bottom, and the guide pins were tightened with the driver (Fig. 2).

#### In the direct acrylic resin-splinted technique

The direct impression copings were tied up with four complete loops of dental floss (REACH® Mint Waxed Floss, Johnson and Johnson Personal Products) using a forceps. Autopolymerizing acrylic resin (Acrostone cold cure special tray material, Cairo, Egypt) was applied around the impression copings using an incremental application technique till the surface of the transfer copings are fully covered with a layer about 2 mm in thickness. A silicone index (Zetaplus, Zhermack SpA, Italy) was made after the first splint for each cast to standardize the amount of acrylic resin used and used as a reference for splinting.

**Fig. 3:**
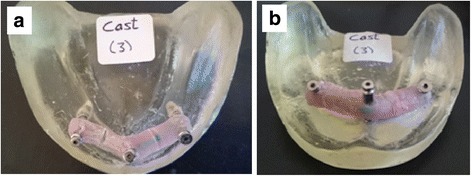
**a** The splint sectioned into three separate pieces. **b** Resplinting with acrylic resin

After 17 min, the splint was sectioned into three pieces with a diamond disk. The impression copings were then resplinted with same acrylic resin (Fig. 3 a, b). Another 17-min interval was allowed after additional splinting to reduce the effects of polymerization shrinkage.

Impressions were inspected and repeated when any inaccuracy was found.

### Cast production procedure

All the impressions were poured with type IV dental stone (elite® stone, Zhermack GmbH Deutschland) using a single prefabricated mold made with laboratory silicone (Ramses medical products factory, Alex, Egypt).

After setting of stone, the casts were separated from the impressions. The three healing abutments were tightened to the implant analogs before the measuring procedures. All casts were labeled and stored at room temperature for 24 h prior to measurements.

### Measurement procedure

A coordinate measuring machine (CMM) (Mitutoyo CRYSTA-Apex S544, Japan) was used to evaluate the positional accuracy of the samples with accuracy of 0.0001 mm.

**Fig. 4:**
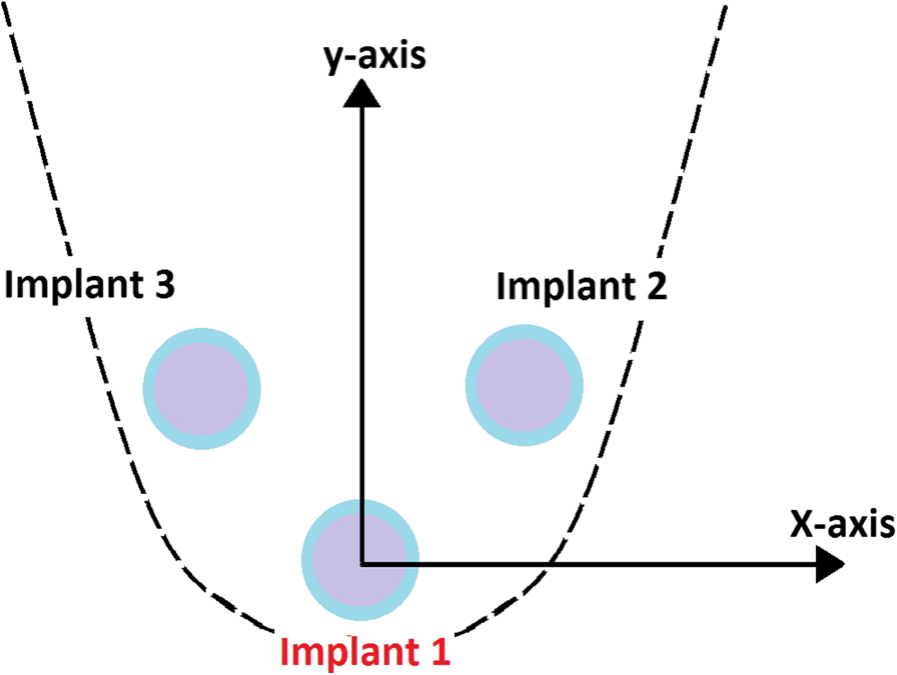
Implant donations

The implant abutments were donated as seen in (Fig. 4).

**Fig. 5:**
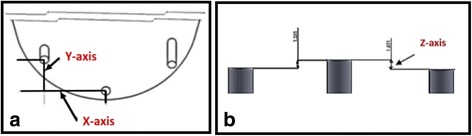
**a** Inter-implant distances in *x-* and *y*-axes. **b** Inter-implant distance in *Z* axis

The center of abutment 1 was considered as the reference point for all measurements. The planar surface from this point was regarded as XY. Two imaginary XZ lines were considered between the centers of the analogs 1, 2 and 1, 3. The XZ planes were perpendicular to XY plane. Therefore, the center of analog 1 was laid on the origin (0, 0, 0). CMM measured the coordinates of each analog with respect to the reference point (Fig. 5).

The center of each implant abutments was located using a CMM probe by touching eight points on the circumference of the outer diameter of the implant abutments.

**Fig. 6:**
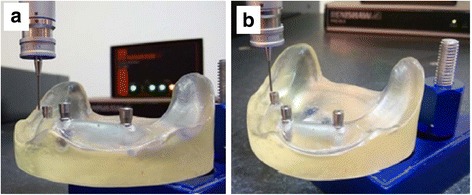
**a** Measuring the outer diameter. **b** Measuring the upper surface

Four points on the upper surface of each implant abutment were measured to form a plane used to calculate the vertical distances between implant abutments 1 and 2, and 1 and 3 in the *z*-axis (Fig. 6).

The distances (in micrometers) between the implant centers with the reference point were calculated according to the following formula [9]:

The distance from the reference point (*r*) = $\sqrt{x^2+{y}^2+{z}^2}$.

Absolute error (Δ*r*) was calculated by comparing the Euclidean distance between the analogs in the duplicated cast with the distance in the definitive cast:

Absolute error Δ*r* = $$ \sqrt{x_{m+}^2}{y}_{m+}^2{z}_m^2-\sqrt{x_{d+}^2}{y}_{d+}^2{z}_d^{2.} $$

where *m* = master and *d* = duplicated.

Each model has two Euclidean distances and named Δ*r*1 (absolute error between implant abutments 1 and 2) and Δ*r*2 (absolute error between implant abutments 1 and 3).

### Statistical analysis

Mean and standard deviation were calculated for each Euclidean distance. One-way ANOVA followed by post hoc tests was performed to detect significance between groups. Statistical analysis was performed using SPSS statistics software for Windows. *P* values ≤ 0.05 were considered to be statistically significant in all tests.

Two-way ANOVA was used to evaluate the influence of different impression techniques and implant angulations on the accuracy of impressions at a significance level of .05 (SPSS version 20, IBM).

## Results

The mean and standard deviation values of each of the two Euclidean distances measured in micrometer (μm) for the nine study groups are presented in Table 1.

**Table 1:** Mean and standard deviation values of each of the two Euclidean distances measured in micrometer (μm) for the study groups

Groups	Subgroups	Mean ± SD	
		Δ*r*1	Δ*r*2
Group 1: Indirect technique *n* = 10 per subgroup	Subgroup A: cast 1	48.11 ± 8.2	54.08 ± 7.2
	Subgroup B: cast 2	64.8 ± 8.2	68.3 ± 8.6
	Subgroup C: cast 3	93.8 ± 4.83	90.9 ± 4.2
Group 2: Direct unsplinted technique *n* = 10 per subgroup	Subgroup A: cast 1	41.4 ± 9	50.7 ± 13.3
	Subgroup B: cast 2	47.39 ± 5.51	55.28 ± 7.3
	Subgroup C: cast 3	67.07 ± 5.7	68.66 ± 4.7
Group 3: Direct splinted technique *n* = 10 per subgroup	Subgroup A: cast 1	40.3 ± 6.7	42.8 ± 8.5
	Subgroup B: cast 2	44.64 ± 4.63	45.58 ± 3.4
	Subgroup C: cast 3	49.96 ± 10.6	50.36 ± 10.3

*Δr1* the absolute error between implant abutments 1 and 2, *Δr2* the absolute error between implant abutments 1 and 3

### Comparing between study groups

#### Effect of impression technique on accuracy of final impression

- *In cast (1*):One-way ANOVA revealed no statistically significant differences in deformation between the three impression techniques (*F* = 2.694, 3.276; *p* value = .085, 0.056 for Δ*r*1 and Δ*r*2, respectively) as shown in Table 2**.**
- In cast (2): One-way ANOVA revealed statistically significant differences in deformation between the three impression techniques (*F* = 30.03, 22.95; *p* value = < .0001^*^ for Δ*r*1 and Δ*r*2, respectively) as shown in Table 3**.** Post hoc test showed that indirect technique was significantly (*p* < 0.05) less accurate than direct unsplinted and direct splinted techniques which were not significantly different from each other (*p* > 0.05) for Δ*r*1 and Δ*r*2.
- In cast (3): One-way ANOVA revealed statistically high significant differences in deformation between the three impression techniques (*F* = 85.65, 83.56 *p* value = < .0001^*^ for Δ*r*1 and Δ*r*2, respectively) as shown in Table 4. Post hoc test showed that indirect technique was significantly (*p* < 0.05) less accurate than direct unsplinted which was significantly (*p* < 0.05) less accurate than direct splinted techniques for Δ*r*1 and Δ*r*2.

**Table 2:** Comparison of impression techniques in cast 1 using one-way ANOVA

	Comparison of impression techniques in cast 1 (parallel condition)			*F*	*p* value
	Group 1 Indirect	Group 2 D.unsplinted	Group 3 D.splinted		
	Mean ± SD	Mean ± SD	Mean ± SD		
Δ*r*1	48.11 ± 8.2	41.4 ± 9	40.3 ± 6.7	2.694	.085
Δ*r*2	54.08 ± 7.2	50.7 ± 13.3	42.8 ± 8.5	3.276	0.056

*Δr1* the absolute error between implant abutments 1 and 2, *Δr2* the absolute error between implant abutments 1 and 3

**Table 3:** Comparison of impression techniques in cast 2 using one-way ANOVA

	Comparison of impression techniques in cast 2 (angulated 15^৹^)			*F*	*p* value
	Group 1 Indirect	Group 2 D.unsplinted	Group 3 D.splinted		
	Mean ± SD	Mean ± SD	Mean ± SD		
Δ*r*1	64.8 ± 8.2	47.39 ± 5.51	44.64 ± 4.63	30.0341	< 0.0001^*^
Δ*r*2	68.3 ± 8.6	55.28 ± 7.3	45.58 ± 3.4	22.9561	< 0.0001^*^

*Δr1* the absolute error between implant abutments 1 and 2, *Δr2* the absolute error between implant abutments 1 and 3

**Table 4:** Comparison of impression techniques in cast 3 using one-way ANOVA

	Comparison of impression techniques in cast 3 (angulated 30^৹^)			*F*	*p* value
	Group 1 Indirect	Group 2 D.unsplinted	Group 3 D.splinted		
	Mean ± SD	Mean ± SD	Mean ± SD		
Δ*r*1	93.8 ± 4.83	67.07 ± 5.7	49.96 ± 10.6	85.6521	*< 0.0001* ^*^
Δ*r*2	90.9 ± 4.2	68.66 ± 4.7	50.36 ± 10.3	83.5686	*< 0.0001* ^*^

*Δr1* the absolute error between implant abutments 1 and 2, *Δr2* the absolute error between implant abutments 1 and 3

#### Effect of implant angulation on accuracy of final impression

##### For (group 1) the indirect technique

One-way ANOVA revealed statistically significant differences in deformation between cast 3(30^৹^), cast 2 (15^৹^), and cast 1(0^৹^) groups (*F* = 100.65, 71.39; *p* value = < .0001^*^ for Δ*r*1 and Δ*r*2, respectively). Post hoc test showed that indirect technique was significantly (*p* < 0.05) less accurate in case of 30^৹^ angulated implants in cast 3 than 15^৹^ angulated implants in cast 2, which was significantly (*p* < 0.05) less accurate than parallel implants in cast 1 for Δ*r*1 and Δ*r*2.

##### For (group 2) direct unsplinted technique

One-way ANOVA revealed statistically significant differences in deformation between cast 3 (30^৹^), cast 2 (15^৹^), and cast 1 (0^৹^) groups (*F* = 36.2, 9.33 *p* value = < 0.0001^*^, 0.0008^*^ for Δ*r*1 and Δ*r*2, respectively). Post hoc test showed that the distortion values of the duplicate casts obtained from cast 3 (30^৹^) was significantly higher than distortion values for cast 1 (0^৹^) and cast 2 (15^৹^) (*p* < 0.05), which were not significantly different from each other (*p* > 0.05) for Δ*r*1 and Δ*r*2.

##### For (group 3) direct splinted technique

One-way ANOVA revealed no statistically significant differences in deformation (Δ*r*1 or Δ*r*2) between cast 3 (30^৹^), cast 2(15^৹^), and cast 1(0^৹^) groups (*F* = 3.14, 2.18; *p* value = .059, .132 for Δ*r*1 and Δ*r*2, respectively).

#### Interaction between variables

A two-way ANOVA was performed to study the effect of impression technique and implant angulation on the accuracy of duplicate casts. The data obtained in this study reveals significant interaction between impression technique and implant angulation (*p* = < .0001^*^) in the two Euclidean distances and that both variables affect the implant impression accuracy as shown in Tables 5 and 6.

**Table 5:** Effect of implant angulation and impression technique on impressions by two-way ANOVA for Δ*r*1

Source of variation	DF	Mean square	*F*	Sig.
Angulation	2	5668.941	104.382	.000^*^
Imp.tech.	2	4562.156	84.003	.000^*^
Angulation * imp.tech.	4	860.574	15.846	*.000* ^*^
Error	81	54.309		
Total	90			
Corrected total	89			

*DF* degree of freedom

*Significant (*p* < 0.05)

**Table 6:** Effect of implant angulation and impression technique on impressions by two-way ANOVA for Δ*r*2

Source of variation	DF	Mean square	*F*	Sig.
Angulation	2	3356.616	50.873	.000^*^
Imp.tech.	2	4557.783	69.077	.000^*^
Angulation * imp.tech.	4	581.127	8.808	*.000* ^*^
Error	81	65.981		
Total	90			
Corrected total	89			

*DF* degree of freedom

*Significant (*p* < 0.05)

## Discussion

An impression that precisely records the 3-dimensional positions of implants is essential to achieve a passively fitting prosthesis [1, 17]. Therefore, comparative accuracy of impression techniques becomes an important issue in consideration of passive fit [8].

In this study, epoxy resin models were used as reference models because they have appropriate elastic modulus for a bone analog material [18]. They were also found to have better stability than plaster models used in other studies [19, 20].

The models were selected to be with no undercuts because undercuts need high removal forces, which can confound the results. Therefore, removing them from the study favors the reliability of the findings [21].

In the present study, the three implants were placed in each reference model with different angulations to simulate common clinical situations that may necessitate placement of angulated implants in lower premolar region. Furthermore, unlike most of previous studies, the implants in this study were also tilted to the mesial side, which better represents clinical conditions [21].

In this study, impressions were made at implant level because it allows for the selection of the most proper abutments and is helpful in situations where angulation of the abutments is difficult to be determined intraorally [19, 22].

The impression material used for this study was polyvinylsiloxane as it exhibits accuracy and adequate rigidity [23]. Medium consistency was more advantageous than putty consistency because the implants used caused a higher level of stress to the impression copings during the impression procedure. Therefore, the use of a more elastic consistency is advantageous in evaluating the effect of splinting impression copings on impression accuracy [19]. In addition, the single-step technique allows the material to record finer details without slumping of the material in the tray, less time-consuming, and simple to perform [24].

Custom trays were utilized because elastomeric materials are more accurate if used in 2 to 3-mm uniform thickness. All the custom trays were perforated to ensure good retention with the trays [25]. Standardization of custom trays was done through modification of reference models with spacer and making stoppers and then making of the duplicate casts from the modified reference models [26].

Self-cure acrylic resin was selected as a splinting material in this study as it is easy to use and it does not require a dry environment [27]. Acrylic resin splint was sectioned and resplinted after 17 min in order to minimize any discrepancies due to polymerization shrinkage. Mojon et al [28] and other studies [19, 29–31] have stated that separation and reuniting of acrylic splint when done 17 min after the setting reaction allows 80% reduction in the effects of polymerization shrinkage. A silicone index was made to standardize the dimensions of the acrylic resin splints for each specimen [19, 32].

A prefabricated mold was used for pouring all impressions to control the setting expansion and standardize the amount of dental stone used [26]. All stone casts were stored at room temperature for 24 h prior to measurements to make sure that they have reached their optimal mechanical properties [19, 26, 33].

Studies comparing the accuracy of implant impression techniques with methods such as micrometers, Vernier calipers, strain gauges, or measuring microscopes could merely carry out two-dimensional measurements [5, 34–36]. However, when the measurements are two dimensional only, relevant information is lost. Therefore, CMM was used as the measuring device in this study because it made three-dimensional evaluation of any distortion possible. When points from different implant casts have a common reference within a coordinate system, the 3D orientation of analogs can be recorded [37].

The results show that there was no significant difference in accuracy between the impression techniques used with parallel implants. The similar accuracy may be due to removal of the custom tray along the same path as the implant angulation. These results are in agreement with several studies showing no difference between the three impression techniques [19, 20, 30, 38–40].

While in the case of 15^°^ angulated implants, direct unsplinted technique and direct acrylic resin-splinted technique exhibited more accuracy compared to indirect technique. This was in agreement with some studies that found that direct impression technique whether splinted or not is significantly more accurate than indirect technique when angulation of implants increased up to 15° [33, 39].

Furthermore, in the case of 30^°^ angulated implants, the direct acrylic resin-splinted technique was significantly more accurate than the direct unsplinted technique, which was significantly more accurate than the indirect technique. This finding is in agreement with several studies, which reported the superiority of the splinted technique over the non-splinted technique for making an impression of angulated internal connection implants [6, 29, 31, 39, 41].

Regarding implant angulation, the results of this study found that increasing implant angulation to 15° or 30° affected the accuracy of indirect impression technique. While in the direct unsplinted technique, no difference in accuracy was found between parallel condition and 15° angulated condition. The increased displacement of impression material and the difficult removal of the impression tray in case of angulated implant were believed to be the source of error in the indirect impression technique. In the direct technique, the impression coping remains in the impression, which reduces the effect of implant angulation and the impression material deformation upon removal from mouth.

These findings agreed with Lee et al. [33] and Carr et al. [42] who found significant difference in accuracy of indirect technique with angulated implants, while no difference in accuracy of direct technique up to 10° and 15° angulations, respectively. Conrad et al. [20] found that angulation of implants up to 15° did not affect the accuracy of both indirect and direct techniques.

In this study, increasing the angulation between implants to 30° affected the accuracy of direct unsplinted technique while it did not affect the accuracy of direct splinted technique significantly. This is in agreement with Tsagkalidis et al. [39] and Martínez-Rus et al. [31]. This may be because splinting the impression copings using a rigid material prevented individual coping movement during the impression making procedure [1].

This study showed that significant interaction existed between impression technique and implant angulations and that both affected implant impression accuracy. As implant angulations increase, distortion in the experimental cast increases. This can be explained with increased material deformation upon impression removal. These results find support in some other studies [39, 43].

According to the recorded data, the null hypothesis was partially rejected because the accuracy of the impression techniques was only different in angulated implant conditions and there was an interaction between impression technique and implant angulations and that both affect impression accuracy.

## Conclusions

The accuracy of definitive casts was affected by the impression technique only in angulated implant conditions where direct splinted technique provided the most accurate position transfer. In parallel implant situation, the three techniques were similar.

When implant angulation increases, the forces of deformation increase which requires an impression technique that allows precise inter-implant relationship. The indirect technique showed the highest distortion values when angulated implants were used followed by direct-unsplinted technique then direct acrylic resin-splinted technique.
